# Swine Model of Thrombotic Caval Occlusion Created by Autologous Thrombus Injection with Assistance of Intra-caval Net Knitting

**DOI:** 10.1038/srep18546

**Published:** 2015-12-18

**Authors:** Wan-Yin Shi, Shuang Wu, Lan-Yue Hu, Chang-Jian Liu, Jian-Ping Gu

**Affiliations:** 1 Department of Interventional Radiology, Nanjing First Hospital, Nanjing Medical University, Nanjing 210006, China; 2 Department of Vascular Surgery, Nanjing Drum Tower Hospital, The Affiliated Hospital of Nanjing University Medical School, Nanjing 210008, China

## Abstract

To evaluate the feasibility of a swine model of thrombotic inferior vena cava (IVC) occlusion (IVCO) created by autologous thrombus injection with assistance of intra-caval net knitting. Sixteen pigs were included and divided into two groups: Group A (n = 10), IVCO model created by knitting a caval net followed by autologous thrombus injection; Group B (n = 6), control model created by knitting a net and normal saline injection. Venography was performed to assess each model and the associated thrombotic occlusion. The vessels were examined histologically to analyse the pathological changes postoperatively. IVCO model was successfully created in 10 animals in Group A (100%). Immediate venography showed extensive clot burden in the IVC. Postoperative venography revealed partial caval occlusion at 7 days, and complete occlusion coupled with collateral vessels at 14 days. Histologically, Group A animals had significantly greater venous wall thickening, with CD163-positive and CD3-positive cell infiltration. Recanalization channels were observed at the margins of the thrombus. By contrast, no thrombotic occlusion of the IVC was observed in Group B. The thrombotic IVCO model can be reliably established in swine. The inflammatory reaction may contribute to the caval thrombus propagation following occlusion.

Deep vein thrombosis (DVT) and pulmonary embolism (PE) constitute venous thromboembolism, a leading cause of mortality and morbidity that affects over 2 million people in the United States each year^1^,^2^,^3^,^4^. If treated inadequately or untreated, thrombosis can propagate over time and cause thrombotic occlusion in the affected veins, usually presenting as chronic venous insufficiency or thrombosis recurrence^5^,^6^. In the setting of thrombotic venous occlusion, achieving vein patency and reconstituting flow is often difficult with current treatment options, thus compromising the quality of life.

Thrombosis is a process involving at least one of three elements of Virchow’s triad. However, the pathways underlying the evolution from thrombosis to venous occlusion are not fully understood^4^. Thus, establishment of new animal models of thrombotic venous occlusion is important for mechanistic and treatment studies. Mouse models, which are widely used in basic research on thrombosis, are not applicable for endovascular treatment studies because of the small calibre of their veins. In the present study, we created a swine model of thrombotic inferior vena cava (IVC) occlusion (IVCO) and evaluate the feasibility of the model with venography and pathological examination.

## Results

### Technical feasibility

No animals died or experienced any procedure-related complications such as major haemorrhage or infection during the study period. No signs of dyspnoea were observed. The IVCO model was achieved in 100% (n = 10) of animals in Group A. There were no sex-based difference in technical success, complications, histopathology, or angiographic findings in either group.

### Angiographic features

**Figure 1:**
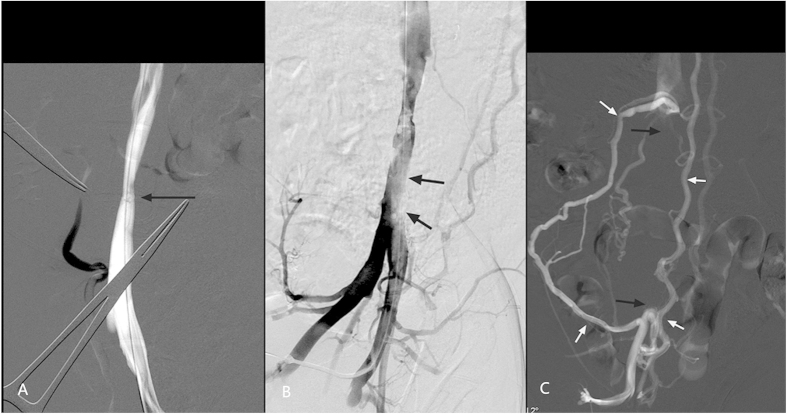
Venography of the IVCO model. (**A**) Immediate venography after knitting showed slight coarctation at the knitting site of knitting (arrow). (**B**) Immediate venography after injection of autologous thrombus showed the sign of ‘filling defect’ in CIV and infrarenal IVC. (**C**) Venography via the right femoral vein was repeated at 14 days after operation and revealed a complete occlusion of the IVC (black arrows). Note that numerous collateral veins were developed (white arrows). IVCO, inferior vena cava occlusion; CIV, common iliac vein; IVC, inferior vena cava.

*Group A.* Venogram of the IVC prior to operation showed normal appearance with a mean diameter of 9.90 ± 0.71 mm. Intraoperative venogram performed immediately after knitting showed slight coarctation at the knitting site and signs of ‘filling defect’ in the infrarenal IVC, indicating thrombus that was captured *in situ* (Fig. 1A,B). The captured thrombus extended above the net. The appearance of the IVC above the level of the renal veins was normal.

Pulmonary arteriography revealed no signs of PE. The venograms repeated at 7 days revealed partial occlusion of the infrarenal IVC and the common iliac veins in all subjects. At 14 days, the infrarenal IVC was completely occluded in five subjects, indicating thrombus propagation over time (Fig. 1C). The collateral vessels were developed, including the pelvic vein plexus, ascending lumbar veins, and inferior epigastric vein. The estimated thrombus volume was 2.98 ± 0.16 cm^3^, 3.33 ± 0.29 cm^3^, and 5.6 ± 0.95 cm^3^ at 0, 7, and 14 days post injection, respectively. The difference in thrombus volume was significant different at 14 days versus 0 days (P = 0.026) and at 14 days versus 7 days (P = 0.017), indicating progression of thrombus size over time. There were no differences in thrombus volume at 0 days and at 7 days (P = 0.178).

*Group B.* The venogram of the IVC prior to operation showed normal appearance with a mean diameter of 10.17 ± 0.74 mm. The intraoperative venogram performed immediately after knitting showed slight coarctation at the knitting site and no signs of captured thrombus. The venograms repeated at 7 and 14 days also revealed no evidences of thrombus. The IVC was maintained patent without visible collateral vessels.

### Manual aspiration and rheolytic thrombectomy for thrombotic IVCO

**Figure 2:**
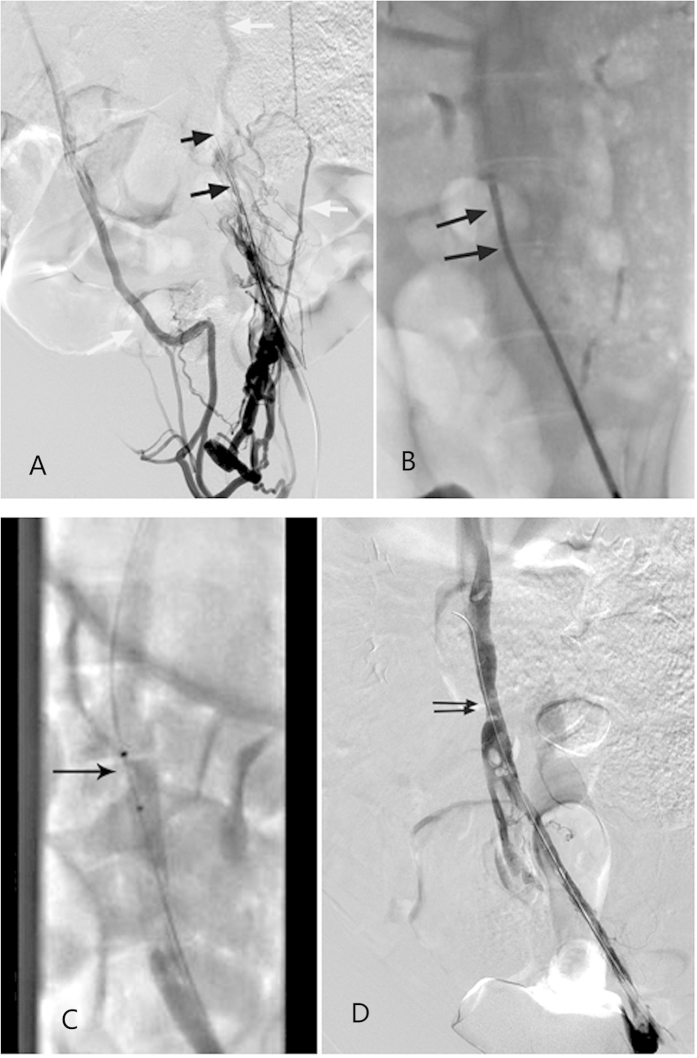
Thrombotic IVCO undergoing manual aspiration and AngioJet rheolytic thrombectomy. (**A**) Venography via the left femoral vein was repeated 2 weeks postoperatively and revealed complete occlusion of the IVC (black arrow). Note that numerous collateral veins were developed (white arrows). (**B**) An 8 French guiding catheter was placed into the IVC to aspirate the thrombus (black arrow). (**C**) Two runs of thrombectomy with an AnjioJet rheolytic device (black arrow) were performed over occluded segment. (**D**) Immediate venography after thrombectomy showed the IVC and left iliac vein were partially reopened, and the blood flow was almost restored. Note the slight coarctation of the IVC, indicating the knitting site (black arrow). IVCO, inferior vena cava occlusion; IVC, inferior vena cava.

Three IVCO models in Group A were successfully treated with manual aspiration and rheolytic thrombectomy. The intra-caval net turned was not an obstacle for manipulating these endovascular devices (Fig. 2A–C). Immediate venography after aspiration and thrombectomy showed partial removal of the thrombus from the iliac vein and the IVC, with successful reconstruction of blood flow in all three subjects (Fig. 2D). The estimated thrombus volume was significantly reduced from 5.88 ± 0.22 cm^3^ prior to endovascular treatment to 0.78 ± 0.19 cm^3^ post treatment (P = 0.002).

### Laboratory testing results

In Group A, the levels of proinflammatory (IL-6, hsCRP) and prothrombotic (D-dimer, fibrinogen, TF, PAI-1) markers were significantly higher at 7 and 14 days post-model compared with levels at 0 days (Tables 1 and 2). However, these markers were not changed in Group B (Tables 1 and 2).

### Histopathology

**Figure 3:**
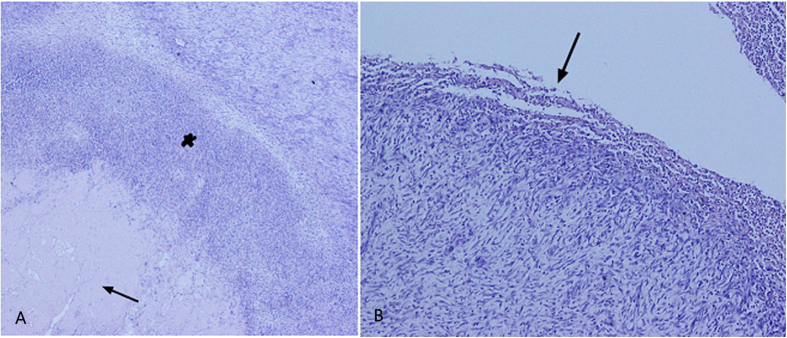
Histological examination of the IVC tissue by H&E staining (×40). (**A**) The IVC tissue obtained at 14 days after operation from the IVCO model showed thickened endothelium with diffuse infiltration of inflammatory cells (asterisk) and a thrombus (arrow) tightly adhered to the vein wall. (**B**) The IVC tissue obtained from the control model showed no evidence of the thrombus. Note the exfoliated endothelium evident at the knitting site (arrow). H&E, haematoxylin and eosin; IVCO, inferior vena cava occlusion; IVC, inferior vena cava.

**Figure 4:**
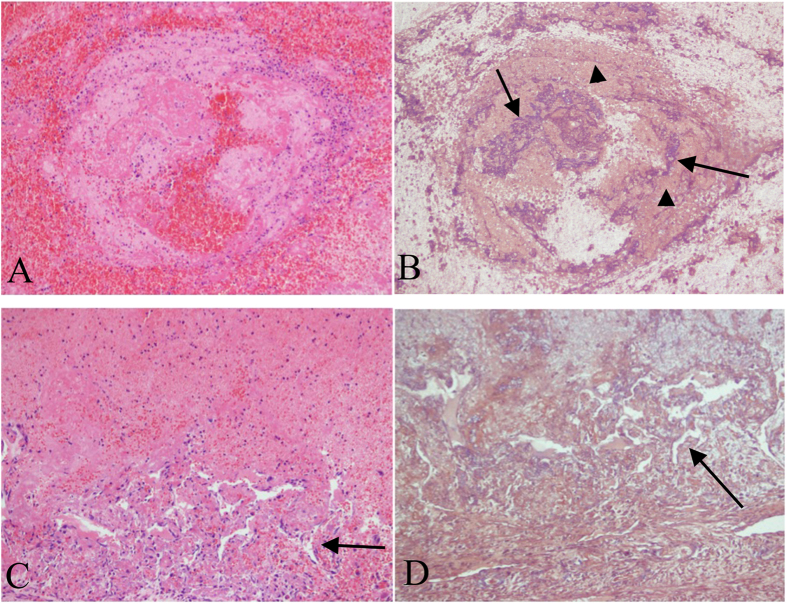
An IVCO model with H&E (×200) and PTAH (×200) staining 14 days post model creation. Mixed thrombus with infiltration of macrophages, neutrophil, and lymphocytes tightly adhered to the thickened caval wall (**A**). Collagen fibers deposition (arrowhead) and fibrosis (arrow) was observed in the thrombus (**B**). H&E (**C**) and PTAH (**D**) staining showed recanalization channels at the margins of the caval thrombus (arrow). H&E, haematoxylin and eosin; IVCO, inferior vena cava occlusion; PTAH, phosphotungstic acid haematoxylin.

In Group A, intraluminal thrombus was observed in all animals and was firmly adhered to the IVC endothelium at 7 days. Histological analysis of the thrombus revealed a mixture of platelets, erythrocytes, and fibrin in varying proportions by light microscopy. Platelet aggregates were most often attached peripherally to the vein wall. Erythrocytes were more randomly distributed. At 14 days, a moderately organized thrombus was observed. The venous endothelium and wall were thickened and showed extensive inflammatory cell infiltration (Fig. 3A). The changes were more apparent and severe at 14 days compared with those at 7 days. In Group B, no animals showed intraluminal thrombus, and the venous endothelium was smooth without any leukocyte infiltration within vein wall (Fig. 3B). Phosphotungstic acid haematoxylin–stained sections showed that collagen fibres were deposited and arranged irregularly in the caval wall and thrombus at 14 days (Fig. 4A,B). Recanalization channels were also observed at the margins of the thrombus (Fig. 4C,D), some of which bridged the caval wall and the thrombus.

**Figure 5:**
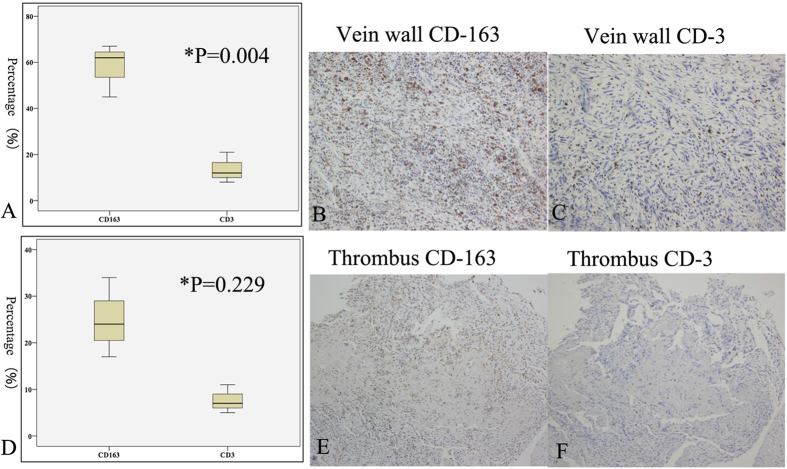
An IVCO model with immunohistochemical staining 14 days post model creation. The indices of CD163-positive and CD3-positive cells that infiltrated in the caval wall were 58.0 ± 6.7% and 13.6 ± 3.8%, respectively (**A**). *P = 0.004 for comparison of CD163-positive and CD3-positive cells. A high number of CD-163 positive cells (brown) infiltrated the vein wall (**B**), while the CD3-positve cells (brown) were distributed sporadically (**C**). (**D**) CD163-positive (25.1% ± 4.9%; **E**) and CD3-positive cells (7.7% ± 1.8%; **F**) that infiltrated in the thrombus were not significantly different (P = 0.229). IVCO, inferior vena cava occlusion.

The caval walls and clots were also immunostained for CD163 and CD3 (Fig. 5). In Group A, CD163-positive cells (monocyte/macrophage) were found infiltrating the caval wall and the thrombus at 7 days (infiltration index, 17.7 ± 3.1% and 6.3 ± 3.1%, respectively), which then significantly increased at 14 days (58.0 ± 6.7% and 25.1 ± 4.9%, respectively) (caval wall, P = 0.004; thrombus, P = 0.024). The index of CD163-positive cells in the caval wall in Group B (1.81 ± 0.98%) was significantly less than that in Group A (P < 0.001).

In Group A, CD3-positive cells (lymphocyte) were also found infiltrating the caval wall and the thrombus at 7 days (2.9 ± 1.8% and 2.8 ± 1.7%, respectively) and at 14 days (13.6 ± 3.8% and 7.7 ± 1.8%, respectively). There were no differences between the index of CD3-positive cells at 7 days and that at 14 days (caval wall, P = 0.054; thrombus, P = 0.071). The index of CD3-positive cells in the caval wall in Group B (0.54 ± 0.72%) was significantly less than Group A (P < 0.001).

## Discussion

In the present study, we established an IVCO model in swine by injecting an autologous thrombus that was captured by an intra-caval knitted net. The entrapped thrombus became a core from which extensive IVC occlusion developed over time. Our data also suggest that inflammatory reactions play an important role in development of the thrombotic occlusion. To our knowledge, this is the first report of an IVCO model created with this technique, without the requirement for surgical ligation or balloon occlusion, a widely-accepted method for creating models of venous thrombosis or thrombotic occlusion.

DVT and PE are relatively common diseases, and disproportionately affect older populations^7^. Acute DVT and PE may progress into chronic disease, manifesting as venous occlusion, chronic venous insufficiency, and chronic pulmonary hypertension. Unlike acute thrombosis, the treatments for chronically thrombotic occlusion are often challenging using surgical conversion or endovascular recanalization. Indeed, the Surgeon General’s Call to Action to Prevent Deep Vein Thrombosis and Pulmonary Embolism^7^ invited multiple stakeholders to work together in a coordinated effort to combat this serious health problem. Thus, creation of ideal animal models is fundamental to our understanding of venous thrombosis and following occlusion.

Models of venous thrombosis or thrombotic occlusion are typically developed in mice or large animals^8^,^9^. Murine models are categorized on the basis of the involved veins^10^. Small vein models can be induced by mechanical injury^11^, endothelial stimulation^12^,^13^, and photochemical injury^14^, while large vein models include the ferric chloride model^15^, IVC ligation model^16^,^17^,^18^, IVC stenosis model^19^,^20^,^21^,^22^, and electrolytic vein model^10^,^23^. The main disadvantage of murine models is their relatively small vessel size, which limits their applicability to fields such as endovascular research.

Non-human primates, pigs, and dogs have a similar venous anatomy to humans, which overcomes some of the disadvantages associated with murine models. The most widely-accepted methods to induce venous thrombosis in large animals are based on flow impairment produced by endovascular balloon occlusion^24^,^25^ or surgical ligation of the targeted vein. The advantages of endovascular approach models include minimal invasiveness and technical safety. However, a duration time of 6 h to several days is usually required for indwelling venous balloon catheters, with potential for procedure-related complications including potential balloon migration, renal failure, and PE^26^. Other drawbacks include the higher cost associated with a catheter balloon and other special endovascular devices, and thrombus instability after balloon removal.

Surgical interruption to induce venous thrombosis usually requires additional ligation of the branches of the targeted vein to promote spontaneous thrombosis, making the procedures more complicated. The advantages of surgical approaches include quantifiable amounts of vein wall tissue and production of a stable and durable thrombus. However, surgical models have a relatively higher mortality. Lack of blood flow due to the venous obstruction may also reduce the maximal efficacy of systemic therapeutic agents on the thrombus and vein wall. Moreover, the interruption of the vein may impair the navigation of some endovascular devices.

Several adjunctive methods, including injection of thrombin^24^,^25^,^27^ or soluble ethanol^28^, are useful for promoting thrombosis and venous occlusion. In the present study, we established a novel IVCO model in swine. We knitted an intra-caval net followed by injection of autologous thrombus, which developed into a venous occlusion over time. This procedure was very simple, and could be accomplished within an hour. Our method has significantly lower costs and reduced complications compared with endovascular models, as catheter balloons and other endovascular devices were not required. No major complications were observed any animals in our study. Further, the intra-caval net allowed guidewires and catheters to pass through the meshes, and did not impair the AngioJet’s manipulation. In our pilot studies, this venous occlusion model also responded well to treatment using mechanical aspiration and thrombectomy.

In the present study, light microscopy examination indicated involvement of an inflammatory component in thrombus propagation. In the IVCO models, the venous wall was thickened with diffuse infiltration of inflammatory cells. There was a high number of CD163-positive cells (monocyte/macrophage) infiltrating the caval walls and the thrombus. These changes were greater at 2 weeks postoperatively, and suggested that the thrombus itself was not inert, but rather may dictate the venous wall response. These data provide support for reports that the longer a DVT is in contact with the vein wall, the greater the damage^29^,^30^.

Inflammation plays a key role in thrombosis development, and reflects an interaction between the thrombosis and the vein wall. We also observed sporadic infiltration of CD3-positive cells (lymphocyte) into the caval walls and the thrombus. Venous thrombi can exhibit fibrillar collagen deposition and recanalization^31^,^32^, while macrophages express a wide range of collagen isoforms^31^. Further, the formation of new endothelial-lined channels in thrombus recanalization may represent a type of angiogenesis^32^. In the present study, the induced thrombus presented similar properties. Given the similarity in the inflammatory process between humans and our IVCO model, we suggest that this model is a useful tool to further our understanding of the mechanisms of venous thrombus following thrombotic occlusion.

A local inflammatory response in the vein wall and activation of the coagulation cascade is associated with the release of proinflammatory factors including IL-6 and CRP, as well coagulation and fibrinolytic system proteins including fibrinogen and PAI-1^1^. In response to cytokines and inflammatory mediators (IL-6, hsCRP), endothelial cells express and show an increased activity of TF or tissue thromboplastin. Thromboplastin is the cellular receptor of circulating factor VII, and their interaction initiates the coagulation cascade^1^,^2^. Once the clots are formed, they may undergo uncontrolled growth, resulting in various degrees of venous obstruction. PAI-1 also plays an active role in this process^1^. In the present study, although the caval clots were not spontaneously formed, we found evidence that similar biochemical markers may play role in the process of propagation^33^,^34^.

There are some potential limitations of the IVCO model. First, the model carries the thrombus, unlike real vein thrombosis in humans, and is therefore not suited to thrombogenesis research. However, real vein venous thrombosis often seems to propagate, as we observed for the IVC thrombosis, existing mainly as an extension from the iliac veins or a complication following caval filter placement. The IVC thrombus can grow over time from its origin, and finally cause chronic IVCO. Our preliminary data also show inflammatory cell infiltration, collagen fibre deposition, and recanalization in our caval thrombus, which are observed in patients with venous thrombosis. Additionally, follow-up venography indicated that the caval obstruction was a result of thrombus propagation. Thus, we believe that our model can duplicate human venous thrombus and its evolution to thrombotic occlusion. Because of the differences in IVC calibres between animals, we could not determine the exact size of the meshes during knitting the intra-caval net. In our experience, four sutures were enough to lodge the injected thrombus effectively in swine, without sacrificing the capacity of endovascular device navigation through the meshes. However, the standard procedure of knitting such a net should be validated further. Finally, endovascular treatment in our IVCO model was beneficial, most likely because the caval thrombus was acute in nature. Further studies should be performed to evaluate the relationship between the thrombus age and various endovascular treatments.

In conclusion, we created a successful IVCO model in swine by injecting an autologous thrombus with assistance of an intra-caval net. This model exhibited a steady progression from thrombus lodging to thrombotic venous occlusion, which may be due to inflammation. We believe that this venous model will be ideal for studying the interaction between the thrombus and vein wall, and can also be used to test various endovascular devices.

## Methods

This study strictly complied with the Guide for the Care and Use of Laboratory Animals. The protocol was approved by the Animal Ethics Committee of Nanjing First Hospital, Nanjing Medical University.

### Animal model

All swine were purchased from our institutional Laboratory Animal Centre. Sixteen Hanford miniature swine were included in the study (eight males, eight females; age 16–20 weeks; weight 15–20 kg). Anaesthesia was induced by administering 300 mg ketamine and 10 mg diazepam intramuscularly. Each subject was then administered a suspension of 100 ml:5 g glucose injection and 20 ml:200 mg propofol via the auricular vein for maintaining general anaesthesia (1 ml/min). All surgical procedures were performed under sterile conditions.

Each animal was placed in a supine position on a digital subtraction angiography table to allow fluoroscopic guidance during the procedure. A 6F introducer sheath (Terumo) was inserted into the left or right femoral vein using the modified Seldinger technique with the guidance of ultrasound. A 4F pigtail catheter (Cook) was then introduced into the iliac vein through the sheath and a venography (Omnipaque 350; GE Healthcare; Shanghai, China) was performed to visualize the iliac vein and the IVC.

### Knitting a net within IVC lumen

After venography, a 0.035-inch guidewire (Terumo) was kept in the IVC through the sheath. The abdomen was incised at midline, and the IVC carefully exposed and isolated. The previously placed guidewire served as a marker of IVC, as it could be detected by fluoroscopy or felt by hand. Two vessel clips were then used to temporarily block the IVC bloodstream. One clip was placed on the IVC just below the renal veins and the other on the IVC just proximal to the bifurcation of the common iliac veins.

**Figure 6:**
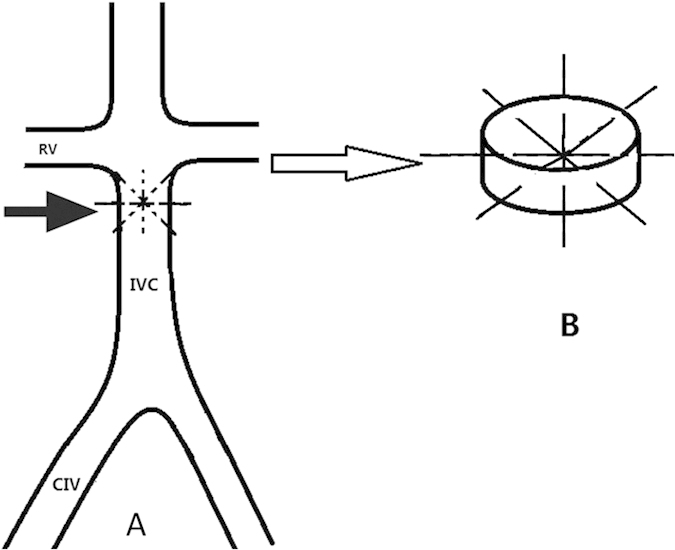
Diagram illustrating the technique for knitting an intra-caval net with sutures. (**A**) Gross visualization of the IVC. Four sutures were passed through infrarenal IVC at different directions (arrow). (**B**) Local visualization of the net. The intra-caval net consisted of four sutures that intercrossed each other at the centre of the IVC lumen. RV, renal vein; IVC, inferior vena cava; CIV, common iliac vein.

Next, four 4-0 polypropylene sutures (PROLENE™; Ethicon, USA) with a needle were used to pass through the IVC (2 cm below the renal veins) individually at anterior-posterior, transverse, and oblique directions (Fig. 6A). The intra-caval net was then made, consisting of four sutures intercrossed at the centre of the IVC lumen (Fig. 6B). Each suture was tied end-to-end at the outside wall of IVC. The net was used to capture the downstream thrombus. The vessel clips were finally removed and repeated venography was performed to inspect the IVC. The abdomen was closed, and the skin was sutured with 4-0 silk.

### Autologous thrombus preparation and administration

All swine with an intra-caval net were divided into two groups: Group A (n = 10; five males, five females) with autologous thrombus injection; Group B (n = 6; three males, three females) with normal saline injection. Blood (10 ml) was collected via the femoral vein using a 20-ml syringe. The blood was then mixed with 500 U lyophilized thrombin powder (Hunan Yige Pharmaceutical Co. Ltd., China) in the syringe for several minutes until it turned into a fresh and soft thrombus. The thrombus was empirically regarded as good for injection if it came out as soft strips when gently pushed by the syringe. The thrombus was administrated manually into the IVC via the 6F sheath. This sheath was placed via the femoral vein with its end at the common iliac vein. The side tube of the sheath was then connected with the syringe. Following injection, the thrombus mainly distributed into the infrarenal IVC that was trapped by knitted net, while a small fraction entered into the iliac vein and the IVC above the net. The thrombus volume in the IVC immediately post injection was calculated, assuming that the thrombus was cylindrical. For all subjects in Group B, 10 ml normal saline was injected.

After injection, venography was performed to inspect the IVC clot burden. A pulmonary arteriography was also performed to inspect the PE via a pigtail catheter placed into the pulmonary artery trunk. Finally, the sheath was removed and the animal was allowed to completely recover and permitted free access to water and food. All animals were administered an antibiotic (cefradine; 10 mg/kg; intramuscular) for 3 days postoperatively.

### Follow-up venography and testing of endovascular devices

A venogram was repeated at 7 and 14 days postoperatively. The procedures were performed under general anaesthesia as previously described. A 6F introducer sheath (Cook) was inserted into the left or right femoral vein, and the contrast agent (Omnipaque 350) was administered intravenously, as described above. Thrombus volume was also estimated at the last venography, assuming that the thrombus was cylindrical.

At 14 days post operation, three IVCO models (two females; one male) were all treated with mechanical thrombectomy (manual aspiration plus AnjioJet rheolytic thrombectomy). First, conventional catheters (such as pigtail and headhunter catheters) and 0.035 inch guidewires were introduced via the sheath into the iliac vein and the IVC to test if they could pass though the thrombus and the knitted net. Aspiration of the thrombus was then manually performed using an 8F guiding catheter (Envoy, Cordis). The AngioJet Rheolytic Thrombectomy System (Boston Scientific, Natick, MA, USA) was then primed following the manufacturer’s instructions. For each model, two runs of rheolytic thrombectomy were performed over the occluded segment. Finally, repeated venography was performed to visualize the outcomes of thrombus removal.

### Laboratory testing

Blood for laboratory testing was collected from the auricular vein of the models. Biochemical testing was performed at 0, 7, and 14 days after model creation. Citrated serum and plasma was stored at −70 °C until assayed. Serum samples were used to determine high-sensitivity C-reactive protein (hsCRP) levels by the nephelometric method (Accute Toshiba) and interleukin (IL)-6 levels by the immunoenzymatic method (Quantikine High Sensitivity IL-6 ELISA KIT; R&D Systems, Inc., MN, USA). Citrated plasma was used to determine D-dimer level by the immunoturbidimetric method (Sysmex CA-7000 System; Siemens), fibrinogen level by the coagulation method (Sysmex CA-7000), plasminogen activator inhibitor-1 (PAI-1) by the immunoenzymatic method (Biopool, Ventura, CA, USA), and tissue factor (TF) using the immunoenzymatic method (Imubind Tissue Factor ELISA KIT; American Diagnostica Inc., USA).

### Tissue sampling and histological analysis

Two female and three male models in Group A and one female and two male models in Group B were euthanized at 7 days post operation, while the remainder were euthanized at 14 days. The IVC between the renal veins and the bifurcation of the common iliac veins was isolated and removed, and immediately fixed in formalin at 4 °C overnight. The tissues were then rinsed with distilled water, dehydrated through graded alcohol solutions, and embedded in paraffin. The venous tissue cross-sections (5 μm) were stained with haematoxylin and eosin (H&E) and phosphotungstic acid haematoxylin (PTAH).

Immunohistochemical staining using anti-CD163 antibody (ab183476; Abcam Inc.) for macrophages, and anti-CD3 antibody (ab16669; Abcam Inc.) for lymphocytes, was performed to assess inflammatory cell filtration in the vein wall and thrombus, defined as the percentage of all cells that were CD163-positive. Image analysis was performed using Image-Pro Plus version 6.0 software in randomly selected vessel fields from each section. A pathologist who reviewed all the specimens and performed the analysis was blinded to animal randomization, treatment procedure, and follow-up protocol.

### Statistical analysis

Data are presented as the range (mean  ±  standard error of the mean). Intra-and inter-group comparisons were performed using the independent-samples t-test. P < 0.05 was considered statistically significant. IBM SPSS for Windows, Version 19.0 (IBM Corp., Armonk, NY, USA) was used to perform statistical analyses.

## Additional Information

**How to cite this article**: Shi, W.-Y. *et al.* Swine Model of Thrombotic Caval Occlusion Created by Autologous Thrombus Injection with Assistance of Intra-caval Net Knitting. *Sci. Rep.* **5**, 18546; doi: 10.1038/srep18546 (2015).

## Figures and Tables

**Table 1:** Comparison of proinflammatory makers between two groups.

Variables	hsCRP (mg/l)			IL-6 (pg/ml)		
	Group A	Group B	P value^†^	Group A	Group B	P value^†^
D0	3.1 ± 0.5 (2.4–3.7)	2.6 ± 0.2 (2.2–2.9)	0.160	3.0 ± 0.2 (2.7–3.4)	2.8 ± 0.2 (2.5–3.0)	0.105
D7	9.6 ± 0.7 (8.9–11.2)	2.8 ± 0.2 (2.5–3.1)	<0.001	6.8 ± 0.3 (6.4–7.2)	3.1 ± 0.2 (2.9–3.4)	<0.001
D14	10.8 ± 1.0 (9.8–12.3)	2.7 ± 0.3 (2.1–3.0)	<0.001	7.1 ± 0.3 (6.9–7.5)	2.9 ± 0.2 (2.8–3.1)	<0.001
P value	<0.001*	0.059		<0.001	0.027	
	<0.001**	0.708		<0.001	0.309	
	0.016***	0.251		0.053	0.282	

All variables are expressed as mean  ±  standard error of the mean (range). *, comparison between D0 and D7. **, comparison between D0 and D14. ***, comparison between D7 and D14. †, comparison between group A and B. P < 0.05 was considered statistically significant. hsCRP, high-sensitivity C-reactive protein; IL-6, interleukin-6; D0, the day at model creation; D7, 7 days post model creation; D14, 14 days post model creation.

**Table 2:** Comparison of prothrombotic markers between the two groups.

Variables	TF (ng/ml)			PAI-1 (ng/ml)			Fibrinogen (g/l)			D-Dimer (μg/l)		
	Group A	Group B	P value^†^	Group A	Group B	P value^†^	Group A	Group B	P value^†^	Group A	Group B	P value^†^
D0	257.2 ± 72.6 (178.3–410.9)	207.8 ± 32.6 (178.9–259.2)	0.141	11.5 ± 3.9 (6.8–20.1)	9.9 ± 1.5 (7.9–11.9)	0.373	3.4 ± 1.0 (1.9–5.4)	3.2 ± 0.8 (2.3–4.3)	0.752	302.1 ± 43.3 (205.2–350.7)	314.8 ± 20.2 (286.6–345.4)	0.513
D7	409.2 ± 121.9 (236.7–589.3)	212.5 ± 17.4 (198.3–215.3)	0.002	25.8 ± 3.5 (20.9–30.9)	10.6 ± 1.5 (8.9–12.8)	<0.001	6.6 ± 0.6 (5.8–7.5)	3.6 ± 0.6 (2.8–4.5)	<0.001	1196.6 ± 155.3 (831.3–1325.9)	303.2 ± 13.1 (276.4–337.3)	<0.001
D14	454.2 ± 76.0 (341.2–512.5)	210.8 ± 21.6 (190.4–233.4)	0.002	28.2 ± 1.3 (20.6–30.1)	10.2 ± 1.6 (8.4–10.9)	<0.001	9.4 ± 1.6 (6.4–11.3)	3.7 ± 0.5 (3.2–4.2)	0.001	1042.2 ± 158.1 (988.7–1339.1)	338.5 ± 33.5 (296.2–318.5)	<0.001
P value	*0.003	0.759		<0.001	0.451		<0.001	0.294		<0.001	0.169	
	**<0.001	0.890		<0.001	0.799		<0.001	0.341		<0.001	0.394	
	***0.468	0.898		0.169	0.726		<0.001	0.872		0.090	0.129	

All variables are expressed as mean ± standard error of the mean. *, comparison between D0 and D7. **, comparison between D0 and D14. ***, comparison between D7 and D14. †, comparison between group A and B. P < 0.05 was considered statistically significant. TF, tissue factor; PAI-1, plasminogen activator inhibitor-1.
